# Quality of life of treatment-seeking transgender adults: A systematic review and meta-analysis

**DOI:** 10.1007/s11154-018-9459-y

**Published:** 2018-08-18

**Authors:** Anna Nobili, Cris Glazebrook, Jon Arcelus

**Affiliations:** 1 Institute of Mental Health, Faculty of Medicine & Health Sciences, University of Nottingham, Room B12, B Floor, Innovation Park, Triumph Road, Nottingham, NG7 2TU UK; 2 Nottingham National Centre for Transgender Health, Nottingham, UK

**Keywords:** Transgender, Quality of life, Mental health quality of life, Sex-related quality of life, Voice-related quality of life, Body image-related quality of life

## Abstract

The study aims to systematically extract and analyse data about Quality of Life (QoL) in the transgender population. A systematic literature search and meta-analysis were conducted using the MEDLINE, EMBASE, PubMed, and PsycINFO databases, up to July 2017. Only English language quantitative studies, in adults, which reported the means for validated QoL measures were included. Random-effect meta-analysis was adopted to pool data and estimate the 95% Confidence Intervals (CI). From 94 potentially relevant articles, 29 studies were included within the review and data extraction for meta-analysis was available in 14 studies. The majority of the studies were cross-sectional, lacked controls and displayed moderate risk of bias. Findings from the systematic review suggested that transgender people display poor QoL, independent of the domain investigated. Pooling across studies showed that transgender people report poorer mental health QoL compared to the general population (−0.78, 95% CI = −1.08 to −0.48, 14 studies). However, meta-analysis in a subgroup of studies looking at QoL in participants who were exclusively post-CHT found no difference in mental health QoL between groups (−0.42, 95% CI = −1.15 to 0.31; 7 studies). There was insufficient data for a pre-treatment subgroup. Evidence suggests that transgender people have lower QoL than the general population. Some evidence suggests that QoL improves post-treatment. Better quality studies that include clearly defined transgender populations, divided by stage of gender affirming treatment and with appropriate matched control groups are needed to draw firmer conclusions.

## Introduction

The term transgender (or trans) describes people whose gender identity differs from the sex they were assigned at birth based on their sexual characteristics, whilst the appellation cisgender refers to any individual who is not transgender and whose gender identity matches the sex assigned at birth [1]. Due to the mismatch between gender identity and sex assigned at birth, many transgender people experience severe distress, generally known as gender dysphoria, which tends to ameliorate following transition to the experienced gender [2].

The process of physical transition consists of different stages. Guidelines for the assessment and treatment of transgender and gender non-conforming people have been developed by the World Professional Association of Transgender Health (WPATH) to facilitate this process (Standards of Care, SOC) [2]. The SOC aims to describe the different treatments that transgender people might wish to undergo, known as Gender Affirming Treatments (GAT), which may include puberty suppression, Cross-sex Hormonal Treatment (CHT), Chest Reconstructive Surgery (CRS) and Gender Affirming Genital Surgeries (GAGSs) [2]. Thus, for the present review, the term ‘treatment’ is also used to describe GAT.

GAT produces bodily changes that impact and alter gender role and its expression by developing secondary sexual characteristics of the experienced gender in order for the body to become more congruent with the gender identity of the individual [2]. These changes might be sufficient to mitigate the gender dysphoric symptoms [2] and hence improve the individual’s QoL. However, not every transgender person requires gender affirming treatment and the dysphoria may improve through gender social role transition only. Thus, treatment might vary depending on the specific needs of the transgender person seeking treatment [2].

Many transgender people, particularly prior to their physical transition, face considerable challenges. These challenges can be physiological (development of some of the secondary sexual characteristics of the sex assigned at birth), social (lack of social support, rejection, discrimination, victimisation, transphobia) [3–12] and psychological (e.g. anxiety, depression, low self-esteem) [3, 13–16]. All these factors have been found to have a negative impact on the quality of life (QoL) of transgender people [17, 18].

QoL is a complex and broad concept. It has been described in different ways, such as the quality of one’s life conditions, one’s satisfaction with life conditions, and as a combination of life’s conditions and satisfaction [19]. De Vries and colleagues [20] defined QoL as the individuals’ perceptions of their life satisfaction and happiness that has an impact on objective and subjective wellbeing. Hence, QoL measures can be considered as a way of quantifying the level of functioning and perceived wellbeing of people’s lives [17]. The concept of QoL encompasses a range of different physical and psychosocial domains. Several factors have been shown to affect QoL in transgender populations, such as presence or absence of depression and psychopathology, transitional status (such as the use of cross sex hormone treatment), levels of social support and perceived discrimination [6, 21–29].

The literature regarding QoL in transgender people mainly focuses on four QoL dimensions: voice-related (vQoL); sex-related QoL; body image-related QoL; and general QoL. Voice-related QoL can be described as the impact that the perception of one’s own voice, in terms of femininity and masculinity, has on the QoL of the individual [30]. This dimension is very important for transgender people, as the pitch of the voice is an important aspect of gender expression and perception [31, 32]. Sex-related QoL is a state of social, physical and mental wellbeing related to sexual life [33]. This concept refers to the sexual functioning and general satisfaction with sexual life [34]. Body image-related QoL stems from the notion that experiencing a positive body image is linked with more satisfactory relationships, sexuality, improved well-being and overall general QoL [35]. Thus, transgender people’s incongruence between gender identity and bodily characteristics could potentially impact their body satisfaction and as a consequence their QoL [35–38]. Finally, general QoL describes the overall satisfaction with life not linked to specific physical health conditions and which includes subcategories linked to aspects of mental, physical, and social life [39].

There are mixed results regarding the QoL in the transgender population. While most of the literature suggests that transgender people have lower QoL compared to the general population [17], which increases once on CHT or post-GAGS [28, 40, 41], other studies have not replicated such findings [42, 43]. These mixed results may be explained by the lack of homogeneity in the population studies, as well as by the different types of QoL and measurements used. For instance, the effect of CHT and genital surgery on the QoL of transgender people when compared to the general population is unclear, as studies often use mixed samples in terms of treatment status and/or focus onto different states of transition. The review carried out by Murad and colleagues [27] suggested that CHT improves QoL, sexual and psychological functioning as well as gender dysphoria; however these findings are based on low quality evidence and the actual impact of both medical and social transitions upon QoL needs to be better understood [44].

Therefore, the primary aim of this this study is to conduct a critical systematic review and meta-analysis of studies of QoL in transgender populations and to explore the range of QoL assessed. The present research also aims to investigate the impact of CHT by exploring QoL in transgender people at different stages of gender transition.

Additionally, as there is a lack of understanding of the QoL domains most relevant to transgender people and of how demographic, psychosocial and treatment-related factors influence those domains, this review specifically aims to assess the different dimensions of QoL in transgender populations and their associated factors.

### Eligibility criteria

Studies were included if they aimed to measure QoL in transgender populations using validated QoL tools. Articles were eligible for inclusion if they reported a mean QoL score for a transgender population and were either written in the English language or had an available translation into English. Both cross-sectional and longitudinal studies were included and there was no restrictions on settings. Studies were excluded from this systematic review if they investigated QoL in transgender children (<18 years) as QoL vary with age [45]. Additionally, articles were excluded if they had fewer than 20 participants as in small studies there is a high risk of selection bias and a lack of statistical power [46, 47]. Where different articles utilised the same database and same measures, the most recent article was taken into consideration and included within the meta-analysis. Qualitative studies, case studies, conference abstracts and review articles were also excluded. See Table 1 for summary of the review’s eligibility process.

**Table 1:** Criteria for inclusion of studies within the review

Category	Criteria
Study population	Transgender people Gender Dysphoria, Transsexualism as well as previous diagnoses according to DSM or ICD, or self-defined as transgender
	LGBT studies only if describing transgender people as separate category All races, ethnicities, and cultural groups
	Adults
Sample size	At least 20 participants
Study settings	All settings
	No exclusion criteria based on research setting
Time period	Published from 1946 to July 2017
Publication criteria	Articles in English
	Articles in peer reviewed journal
Study design	Observational studies using standardised measure of QoL. Cross-sectional or longitudinal designs

### Search strategy

PRISMA guidelines were followed [48] to carry out this review. Ovid (PubMed, EMBASE, PsycINFO) and Medline databases were searched from 1946 to July 2017. Terms for transgender people (Transgender, Transsexual, Gender Identity Disorders, and Gender Dysphoria) were searched using the OR function and combined with the terms related to (Quality of Life, QoL, Life Satisfaction) using the “AND” operator. Additionally, the reference lists of pertinent articles were searched to identify any further potential relevant papers.

### Quality assessment

Risk of bias was assessed using an instrument adapted from Ibrahim et al. [49] as this instrument covered the most relevant criteria to assess risk of bias in descriptive studies. Criteria were [1] a clear definition of the target population, [2] adoption of either random, complete or consecutive recruitment or an attempt at recruiting every participant in the sampling frame, [20] sample as representative of the target population or the report presents evidence that results can be generalised to transgender people, acknowledging that most studies included treatment-seeking transgender people attending gender clinics [3] response rate equal or greater than 70%, [4] adequate sample size with a minimum of 300 participants as smaller sample sizes produce large confidence intervals and less precise results [50, 51] and [5] use of validated measures. The chosen criteria were evaluated as providing either a risk of bias (or unclear risk of bias) (1 point) or no risk of bias (0 point). Scores are then summed and an overall risk of bias rating is created where higher scores indicate greater risk of bias. Studies were rated as low risk of bias (++) (when all or most of the criteria were satisfied), moderate risk of bias (+) (when some of the criteria were satisfied) or high risk of bias (−) (when either a few or no criteria were satisfied), as per the NICE [52] guidelines for risk of bias assessment.

### Data extraction

A data extraction table was used to record authors, date of publication, country where the study was conducted, participants’ information (sample sizes, mean age of sample at assessments), information on treatment status, study design, control group and follow-up (if applicable), QoL measures used, results, factors associated with QoL and conclusions. Separate tables were constructed differentiating depending on the QoL domain investigated.

### Meta-analysis

Mental health-related QoL was used as the outcome of interest for the meta-analysis, as it was the most widely reported outcome and physical QoL is more sensitive to the effects of age [53]. The most frequently used QoL measures (e.g. SF-36, SF-12) do not calculate a total score but calculate separate composite scores for mental and physical health. Generic (i.e. not condition specific) mental health-QoL scores for all samples with means and Standard Deviations (SDs) reported were eligible for inclusion in the meta-analysis. When the means and SDs for a cisgender group were provided, these were used as the comparison in the meta-analysis. Where these were not available, normative data most applicable to the study country were obtained from the articles providing validation of the specific measures adopted and were used as comparison. Utilisation of normative data as a control might cause methodological concerns, as this might increase effect sizes; however, to not lose valuable data and to be able to carry out the meta-analyses, this method was deemed as the best approach. This approach was adopted for four studies [54–57].

In longitudinal studies, data from the first time point at which the participants met the age criterion for the review were used. Where studies reported incomplete results, values were either manually calculated (e.g. SDs from means) or authors were contacted to provide the missing data.

A second meta-analysis with a sub-group of studies reporting data for samples of participants who were exclusively post-GAGS, and therefore post-CHT, as the big majority of people undergoing gender affirming surgeries are already on hormonal treatment, was conducted. Pre-treatment-QoL was not assessed due to a lack of studies using exclusively pre-treatment samples. RevMan 5 [58] was utilised to conduct the meta-analyses.

It was hypothesised that the results would be heterogeneous because of differences between studies in the stages of transition investigated (e.g. mixed samples, pre-CHT, post-CHT, post-GAGS), in the diverse types of recruitment utilised (e.g. consecutive, snowballing), in the presence of clinical and/or non-clinical individuals within the samples as well as in the focus onto the different gender identities of the participants (e.g. transman, transwoman, both). Consequently, Random Effects Models (RAM) with 95% confidence interval was used for the analyses as it implies that the selected studies are carried out in diverse populations [59]. *I*^*2*^ statistics were calculated to examine heterogeneity, which is expressed in percentages suggesting different degrees of heterogeneity with 25% indicating low, 50% moderate and above 75% high [60]. Additionally, *Q* statistics were calculated to determine the statistical significance of heterogeneity [61].

### QoL measures used in the review

See Table 2 for a description of the measures used in the studies to assess QoL. Voice-related QoL was assessed using the Voice Handicap Inventory (VHI) and the Transgender Self-Evaluation Questionnaire (TSEQ), sex-related QoL using the sexual subdomain of the WHOQOL-100 and the King’s Health Questionnaire (KHQ), body image-related QoL using the body image-related subdomain of the WHOQOL-100 as well as the Body Image Quality of Life Inventory (BIQLI), and generic (non-condition specific) QoL was measured using the Short Form 36 Health Survey (SF-36), version 2 of the Short Form 36 Health Survey (SF-36-v2), version 2 of the Short Form 12 Health Survey SF-12-v2, WHOQOL-100, WHOQOL-BREF, WHOQOL-BREF-TR or the Subjective Quality of Life Analysis (SQUALA). See Table 2 for a description of the measures.

**Table 2:** Quality of life measures used in the review

Measure	Details
1. Short Form 36 Health Survey SF-36 [62, 63]	This tool was developed to measure multiple operational health indicators of QoL [62]. It is a well-validated international measure of health-related QoL consisting of 36-items providing scores for two summary components (Physical and Mental), which encompass 4 subdomains each. The Physical component includes Physical functioning, Role limitations related to Physical problems, Body pain, whilst the Mental component comprises of Perception of General health, Vitality, Social functioning, Role limitations due to Emotional problems, and Mental health. The scores range from a minimum of 0 until a maximum of 100, where higher scores indicate greater functioning and enhanced perception of QoL. The cut-off for the population norm is around 50. The measure was validated in a wide variety of clinical and non-clinical populations, and it displayed an internal consistency value of .88 when used with Transgender populations [63]. This tool was employed by six studies reported on within this review [43, 54, 55, 64–66].
2. Short Form 36 Health Survey Version 2 SF-36v2 [67]	This measure was developed out of the SF-36. It includes more up-to-date norms and QoL domains. It is a standardised, comprehensive and validated QoL measure assessing two summary scores (Physical and Mental components), which encompass 4 subdomains each. The Physical component includes Physical functioning, Role-physical, Bodily pain, General health, whilst the Mental component comprises of Vitality, Social functioning, Role-emotional, and Mental health. It uses a 5-points Likert-scale ranging from 1 (poor/true) to 5 (excellent/false). Higher scores represent higher perceived QoL levels. This measure has also been used and corroborated in an online sample of transgender men displaying a Cronbach’s alpha for reliability ranging from .93 to .95 [17]. This tool was employed by seven studies reported on within this review [17, 22, 42, 68–71].
3. Short Form 12 Health Survey Version 2 SF-12v2 [72]	This instrument is a subset of the SF-36. It comprises of two summary component scores (Physical and Mental), which encompass 4 subdomains each. The former component includes Physical functioning, Role-physical, Bodily pain, General health, whilst the latter component refers to Vitality, Social functioning, Role-emotional, and Mental health. This measure utilises a 5-points Likert-scale ranging from 1 (poor/true) to 5 (excellent/false). Scores range from 1 to 100, with higher perceived QoL represented by higher scores. This measure was validated and showed a good internal consistency, with Cronbach’s alphas of .89 for the Physical component summary and of .86 for the Mental component summary [72]. This tool was employed by one study reported on within this review [32].
4. WHOQOL-100 [39]	It is a self-administered, self-rated measure to assess QoL developed by the World Health Organization QoL group. It has been developed cross-culturally and it maintains excellent psychometric properties and internal consistency. This tool comprises a total of 100-items; 96 measures 24 specific QoL facets, whilst the remaining 4-items estimate General QoL and Overall QoL. The facets are distributed across 6 domains, such as Physical health, Psychological health, Independence, Social relationships, Environment, and Spirituality/Religion/Personal beliefs. In order to investigate the Sexual QoL the specific Sexual activity facet was measured, whilst to examine Body image-related QoL the body image facet was assessed. Items are rated on a 5-points Likert-scale ranging from 1 (very poor/very dissatisfied/not at all) to 5 (very good/very satisfied/extremely). Higher scores indicate greater reported QoL. The scale’s internal consistency values have been found to range between 0.65 and 0.93 [39]. This tool was employed by four studies within this review [34, 56, 73, 74].
5. WHOQOL-BREF [45]	It is a self-rated measure that has been validated in field studies involving approximately 30 languages [27]. It is an abbreviated version of the WHOQOL-100. This tool has 26-items and uses a 5-points Likert-scale measuring 4 domains (Physical, Psychological, Social relationships, and Environment). In addition, there are two questions regarding General QoL and General health. Higher scores indicate greater QoL. Internal consistency values cross-culturally have been found ranging from .51 to .89 [45]. This tool was employed by three studies within this review [20, 21, 57].
6. WHOQOL-BREF-TR [75]	The WHOQOL-BREF-TR is a 27-items 5-point Likert-scale measuring four domains (Physical, Mental, Social and Environmental) in two categories (Perceived QoL in general and perceived health status). It displays acceptable psychometric properties when used on the Turkish population (Cronbach’s alpha ranging from .53 to .83) [75]. This is the Turkish version of the WHOQOL-BREF and it was used by one study included in this review [76].
7. Subjective Quality of Life Analysis SQUALA [77]	It is a self-administered, self-rated, multidimensional QoL measure. It covers 23 QoL domains (e.g. Mental well-being, Perceived health, Physical autonomy, Social relations, Environment) as well as general QoL-related concepts (e.g. justice, freedom, truth, beauty and politics), which identify internal and external reality of everyday life [78]. The measures’ items need to be rated in importance and satisfaction by the person and higher scores indicate better QoL. Cronbach’s alpha was not available. This measure was utilised by one study included within this review [23].
8. King’s Health Questionnaire KHQ [79]	This is a validated measure used to assess QoL, and with the aid of specific questions it is often used to estimate levels of incontinence related-QoL. This is a 29-items Likert-scale assessing ten domains (general health, physical limitations, personal limitations, social limitations, role limitations, personal relationships, emotion, symptom severity, sleep/energy and incontinence) and two categories (QoL and Limitation of daily life). The QoL category is measured with 20-items using a 4-points Likert-scale ranging from 1 (not at all) to 4 (a lot), whilst the incontinence category is measured with 9 items ranging from 1 (a little) to 3 (a lot). A change of 5 points is considered to be significant. It has been validated on a sample of urinary incontinent women with Cronbach’s alpha values ranging from .73 to .89 [80]. This tool was employed by one study reported on within this review [81].
9. Voice Handicap Inventory VHI [82]	This is a validated measure used to self-assess the QoL related to the relative impact of a person’s voice upon daily activities. It is also used to measure QoL of transgender people concerning the impact and influence of their voices. The VHI is a 30-items 5-points Likert-scale ranging from 0 (never) to 4 (always). The items are regularly divided within three domains; functional (F), emotional (E) and Physical (P). The total score (T) is achieved by summing up E, F and P, and it ranges from 0 (normal voice) to 120 (severely affected voice). Scores below 40 represent either mild or absent disability, values between 40 and 60 reflect moderate disability, whilst scores above 60 represent disability. Internal consistency value was found to be .95 [82] This measure has been employed by four studies reported on within this review [24, 30, 31, 83].
9.Transgender Self-Evaluation Questionnaire TSEQ [84]	This is a standardised, subjective measure of voice handicap and vQoL specifically developed for transgender people. It is based on the VHI but adapted to the specific concerns of transgender individuals, such as the impact of masculinity/femininity of voice. It is a 30-items self-reported 5-points Likert-scale ranging from 1 to 5. A total score ranging from 30 to 150 is calculated by adding up the 30 items’ scores and lower scores reflect greater vQoL. The TSEQ was found to have good test-retest reliability (*r* = .97) [85]. Cronbach’s alpha was not available. This tool was adopted by two studies reported on within this review [24, 30].
11. Body Image Quality of Life Inventory BIQLI [86]	This is a 19-items 7-points Likert-scale ranging from −3 (very negative effect) to +3 (very positive effect) that assesses body image-related effects onto 19 different areas of life including sexuality and emotional well-being. Higher scores imply better body image-related QoL. Internal consistency was found to be excellent (α = .95). This tool was used by one study included in this review [35].

## Results

**Fig. 1:**
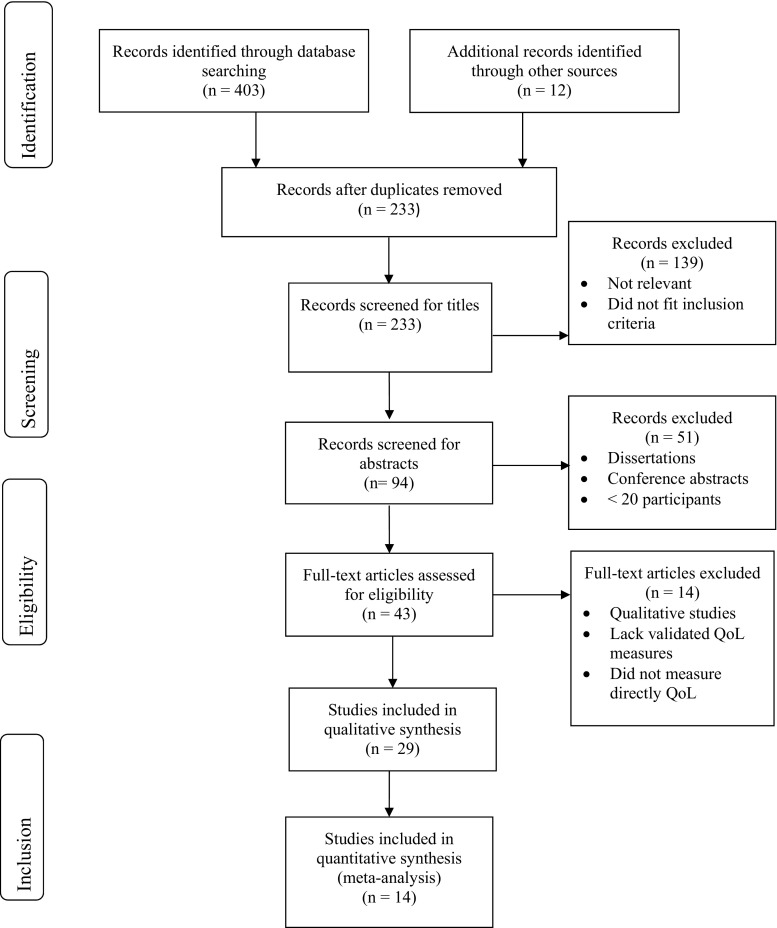
Process of identification of eligible studies for inclusion within the review

A total of 403 studies were identified through database searches, 288 through Ovid and 115 through PubMed. An additional 12 articles were selected for inclusion in the review after screening reference lists of relevant papers. After removing duplicates, 94 abstracts were screened by the first researcher (AN), which resulted in 43 studies that were read in full. Of these, fifteen were excluded due to reasons such as lack of a validated QoL measure (*n* = 4), of direct measurement of QoL (*n* = 6), of results reported specifically for transgender people (n = 4) and one study was qualitative. Finally, a sample of 29 papers was discussed, agreed with the other researchers (JA and CG) and included within this review. See Fig. 1 for description of the study’s selection process.

### Study characteristics

The earliest articles included within this review were published in 2006 [17, 87] whilst the most recent papers were published in 2017 [31, 32, 55, 57, 64].

The majority of the studies were conducted in European countries (*n* = 20). Three studies were carried out in Spain [21, 32, 34], in France [22, 23, 66] and in Belgium [43, 65, 83]. Two studies were conducted in Italy [73, 74], UK [69, 70], the Netherlands [20, 35] and Germany [31, 55], whilst one study was carried out in Switzerland [81], one in Sweden [64] and one in Turkey [76]. With regard to non-European countries one study was from Brazil [56], one from China [54] and the remaining articles were from the USA (*n* = 7).

Out of the 29 included articles; a) four explored vQoL [24, 30, 31, 83], b) four looked at sex-related QoL [34, 73, 74, 81], c) three assessed body image-related QoL [35, 73, 74], and e) 22 studies measured generic (non-condition specific) QoL [2, 17, 21–23, 42, 43, 54–57, 64–66, 68–71, 73, 74, 76, 81]. With regard to vQoL, the study by Mora and colleagues [32] measured vQoL with the aid of a non-validated measure as well as general QoL with a well-validated tool, thus the article was included in the subgroup of general QoL. The study conducted by Parola and colleagues [66] was excluded from the sex-related QoL domain, as it did not employ a validated measure to assess sex-related QoL. Studies reporting generic-QoL that either separated mental and psychological subscales or provided a total QoL score (e.g. Castellano et al. – 71) were included in the systematic review. Of the four papers that measured sex-related QoL, three used the sex-related facet of the WHOQOL-100 [34, 73, 74] whilst one paper measured QoL related to incontinency in transgender women post-GAGS and was included within the sex-related QoL domain [81]. Finally, with regard to body image-related QoL, one article used a specific body image-related QoL measure (BIQLI) [35] whilst the others used the body image-facet of the WHOQOL-100 [73, 74].

In terms of study design, 22 studies were cross-sectional [17, 21–24, 30, 34, 42, 43, 54, 55, 57, 65, 66, 68–71, 73, 76, 81, 83] and seven were longitudinal [20, 31, 32, 35, 56, 64, 74], although three of the longitudinal studies [20, 31, 32] only reported cross-sectional data for QoL. Of the 29 included studies, eight compared scores of transgender people to normative data [17, 21, 34, 42, 43, 64, 65, 68], and eight compared transgender to cisgender individuals [21, 22, 30, 31, 69, 70, 74, 81] of which four studies used a matched comparison group [22, 69, 70, 74]. However for one matched study [70] the gender identity of the comparison group was unclear. Four articles compared QoL in transgender women to QoL in transgender men [23, 66, 76, 83]. The majority of studies (*n* = 23) recruited transgender people through clinical services [20–23, 30–32, 34, 35, 43, 55, 56, 64–66, 68–70, 73, 74, 76, 81, 83]. The remaining five studies recruited participants through opportunity sampling, word of mouth, flyers, advertisement and through community outreach [17, 42, 54, 57, 71].

### Risk of bias

Risk of bias was evaluated for the 29 studies according to the criteria stated in Table 1. Only three studies recruited more than 300 participants [17, 42, 71] and the majority either reported response rates lower than 70% or did not mention this information (*n* = 21) thus increasing the risk of sampling bias [17, 20, 23, 24, 30–32, 34, 35, 38, 42, 43, 54, 55, 64, 65, 68, 70, 74, 81, 83]. Overall, only two studies were rated having a low risk of bias [22, 69], twenty studies had a moderate risk of bias [17, 20, 21, 23, 34, 35, 42, 54–57, 64, 65, 70, 71, 73, 74, 76, 81, 83] and seven a high risk of bias [24, 30–32, 43, 66, 68]. See Table 3 for details regarding studies’ quality assessment and risks of bias.

**Table 3:** Risk of bias of studies included in the review

Source	Sample definition (Inclusion criteria)	Recruitment (Random, complete, consecutive)	Representativeness of Sample (Exclusion criteria and clinical/non-clinical populations)	Response rate (min 70%)	Sample Size (min 300)	Comparison	Use of validated measures	Quality rating
1. Auer et al. (2017) [55]	0	1	0	1	1	1	0	+
2. Ainsworth & Spiegel (2010) [68]	1	1	1	1	1	1	0	–
3. Bartolucci et al. (2015) [34]	0	0	1	0	1	1	0	+
4. Başar et al. (2016) [76]	0	0	1	0	1	1	0	+
5. Bouman et al. (2016) [69] UK	0	1	0	0	1	0	0	++
6. Cardoso da Silva et al. (2016) [56]	0	0	1	0	1	1	0	+
7. Castellano et al. (2015) [73]	0	1	1	0	1	0	0	+
8. Colton Meier et al. (2011) [71]	1	1	0	1	0	1	0	+
9. Colton Meier et al. (2013) [42]	1	1	0	1	0	1	0	+
10. Davey et al. (2014) [70]	0	1	1	1	1	0	0	+
11. de Vries et al. (2014) [20]	0	0	1	1	1	1	0	+
12. Gomez-Gil et al. (2014) [21]	0	0	1	0	1	1	0	+
13. Gorin-Lazard et al. (2012) [22]	0	0	1	0	1	0	0	++
14. Gorin-Lazard et al. (2013) [23]	0	0	1	1	1	0	0	+
15. Hancock et al. (2011) [30]	0	1	1	1	1	1	0	–
16. Hancock et al. (2016) [24]	1	1	0	1	1	1	0	–
17. Hoy-Ellis et al. (2017) [57] USA	0	1	1	0	1	1	0	+
18. Kuhn et al. (2009) [81]	0	0	1	1	1	1	0	+
19. Lindqvist et al. (2017) [64] Sweden	1	0	0	1	1	0	0	+
20. Manieri et al. (2014) [74]	0	0	1	1	1	1	0	+
21. Meister et al. (2017) [31] Germany	1	0	1	1	1	1	0	–
22. Mora et al. (2017) [32] Spain	1	0	1	1	1	1	0	–
23. Motmans et al. (2012) [65]	0	0	1	1	1	1	0	+
24. Newfield et al. (2006) [17]	1	1	0	1	0	1	0	+
25. Parola et al. (2011) [66]	0	1	1	1	1	1	0	–
26. T’Sjoen et al. (2006) [83]	0	0	1	1	1	1	0	+
27. van de Grift et al. (2016) [35]	0	0	1	1	1	1	0	+
28. Wierckx et al. (2011) [43]	0	1	1	1	1	1	0	–
29. Yang et al. (2016) [54]	0	1	0	1	1	1	0	+

0 = No risk of Bias; 1 = Risk of Bias; ++ = Low Risk of bias; + = Moderate Risk of Bias; − = High Risk of Bias

## Results of the literature review

### Voice-related QoL

Of the four papers describing vQoL, three used a cross-sectional design [24, 30, 83], whilst one article used a longitudinal design with cross-sectional data for vQoL [31]. There were no studies looking at pre-treatment transgender people. Only one study offered comparisons of transgender people post-treatment with normative data and reported worse vQoL for transgender people when compared to controls [31]. The cross-sectional studies that looked at people post-treatment (GAGS, CHT, Voice Feminisation Treatment - VFT) found transgender people to experience voice-related disability, in that they feel handicapped in everyday life because of their voice [31, 83]. This could be due to the fact that hormone therapy for transgender women had not affected on their voice. Only one study compared people according to their gender identity; this study by T’Sjoen and colleagues [83] found that transgender men report better vQoL compared to transgender women post-GAT. Overall, vQoL appears to be worse in transgender people, particularly in women, even post-GAT. See Table 4 for details.

**Table 4:** Studies investigating voice-related quality of life in transgender people (*n* = 4)

Authors (year) Country	Number of Trans participants, mean age at assessment	Treatment status	Study design	Comparative groups, follow-up	Outcome measures	Results	Factors associated	Conclusions
Hancock et al. (2011) [30] USA	20 TW 48.8 yrs	Post-VFT 100% Post-GAGS 45%	Single centre Clinical group Cross-sectional	CG1 Speakers: 5 cis women (46.8 yrs) 5 cis men (40.8 yrs); CG2 Listeners: 12 cis men (18.8 yrs) 13 cis women (19.65 yrs) (No follow-up)	TSEQ	Self-ratings: Femininity = 529 Likability = 552 Listener ratings: Femininity = 493 Likability = 533	None studied	For TW vQoL moderately correlated with how others perceive their voice. vQoL correlated more strongly with speaker’s perception of voice compared with others’ perceptions
Hancock (2016) [24] USA	81 TW 43 yrs	VFT 46%	Clinical and non-clinical group Cross-sectional	Online vs. paper Completed VHI vs. completed VHI + TSEQ (No follow-up)	VHI TSEQ	General: VHI = 37.5 TSEQ = 76.5 VHI + TSEQ: VHI = 37.6 TSEQ = 76.5	-vQoL: Increased age Femininity of voice	TW reported a wide range of vQoL; some individuals are severely affected by their voices whilst others are not.
Meister et al. (2017) [31] Germany	T0 21 TW 42.1 yrs. T1 18 TW 46 yrs	T0 = Pre-VFT 100% T1 = Post-VFT 100%	Single centre Clinical group Prospective longitudinal with cross-sectional data regarding VHI	T0 vs T1 German control group	VHI	VHI_mea*n*_ = 32.29	None studied	Despite the elevation of vocal pitch, elevated VHI scores indicate transwomen feel handicapped in everyday life because of their voice
T’Sjoen et al. (2006) [83] Belgium	28 TW 20 TM 33 yrs. TW 49 yrs. TM	GAGS 100% CHT 100%	Single centre Clinical group Cross-sectional	TW vs. TM (No follow-up)	VHI	TM: Total = 4(0–10) (F = 1, E = 0, *P* = 3, Phone = 0) TW: Total = 12 (6–31) (F = 1, E = 2, *P* = 6, Phone = 2)	TM + vQoL: Lower DHT Higher LH	Better vQoL for both TW and TM above the cut-off for disability, meaning that they do experience voice-related disability

*CG* Control Group, *CHT* Cross-sex Hormonal Treatment, *Cis* Cisgender, *DHT* Dihydrotestosterone, *E* Emotional, *F* Functional, *FFS* Face Feminisation Surgery, *GAGS* Gender Confirming Genital Surgery, *LH* Luteinizing Hormone, *P* Physical, *TM* Transgender men, *TW* Transgender women, *VFT* Voice Feminisation Treatment

The few studies investigating predictors of vQoL found increased age of the transgender individual, increased femininity of the voice [24] and low dihydrotestosterone as well as high Luteinising Hormone (LH) in the blood [83] to be factors predictive of a positive vQoL in populations of transgender women.

### Sex-related QoL

Four studies investigated sex-related QoL by adopting a cross-sectional design [34, 73, 74, 81]; one offered comparisons with normative data [34], one compared the transgender group with a cisgender group matched for experienced gender [73], one carried out comparisons between transgender men and transgender women as well as between pre- and post-CHT [74] and the fourth study compared QoL linked to incontinence in transgender people post-GAGS to twenty members of the clinical staff who underwent at least one previous abdominal or pelvic operation [81]. This last study was included within this section as it is linked to surgery outcomes and to satisfaction with sexual life.

Only one study looked at a transgender sample pre-GAGS and found that transgender people report worse sex-related QoL than the general population [34]. Studies including people post-GAGS suggested that transgender people still experienced lower sex-related QoL than their matched controls [73, 81]. When looking at gender differences, Castellano et al. [73] suggested that, at post-GAGS, transgender women did not display significantly different sex-related QoL compared to cisgender women, whilst transgender men showed lower sex-related QoL than cisgender men. Instead, studies comparing transgender people according to gender identity reported a significantly lower sex-related QoL in transgender men when compare to transgender women, independently of the transitional status [34, 73]. Only one study described changes in sex-related QoL using a longitudinal methodology [74]. This study found a significant improvement in sex-related QoL for both transgender men and transgender women post-CHT [74]. Overall, it appears that sex-related QoL improves post-GAT. However, such appears to be poor, particularly in transgender men when compared to cisgender men. See Table 5 for details.

**Table 5:** Studies investigating sex-related quality of life in transgender people (*n* = 4)

Authors (year) Country	Number of Trans participants, mean age at assessment	Treatment status	Study design	Comparative groups, follow-up	Outcome measures	Results	Factors associated	Conclusions
Bartolucci et al. (2015) [34] Spain	67 TW 36 TM DSM-IV-TR 31.46 yrs. TW 28.69 yrs. TM	Pre-GAGS 100% CHT 40% (TW 46% TM 28%) Post-CRS 30% (TW 35% TM 19%)	Single centre Clinical group Cross-sectional	Normative data (No follow-up)	WHOQOL-100	sQoL TW: Poor/very poor 48% Good 23% Very good 20% TM: Poor/very poor 54% Good 27% Very good 28%	+ sQoL: CHT Having a partner Less negative feelings	Pre-GAGS about half of trans sample perceived sexual QoL as either poor or very poor compared to the control group
Castellano et al. (2015) [73] Italy	46 TW 14 TM 32.7 yrs. TW 30.2 yrs. TM	+ 2 years post-GAGS 100% CHT 100%	Single centre Clinical group Cross-sectional	60 matched cis control sample (No follow-up)	WHOQOL-100	sQoL TW = 65.85 TM = 54.21	+QoL: Lower LH	Trans people reported levels of QoL similar to cis controls
Kuhn et al. (2009) [81] Switzerland	52 TW 3 TM 51 yrs. Trans	CHT 100% GAGS 100%	Single centre Clinical group Cross-sectional	20 healthy female medical staff, not matched (No follow-up)	KHQ	KHQ = 27.31	None studied	15-years post-GAGS QoL is lower for trans people in domains of General health, Role, Physical and Personal limitation than the cis control group
Manieri et al. (2014) [74] Italy	56 TW 27 TM 32.7 yrs. TW 30.2 yrs. TM	T0 = initiation of CHT 100% T1 = 3 months post-CHT 100% T2 = 6 months post-CHT 100% T3 = 9 months post-CHT 100% T4 = 1 year post-CHT 100%	Single centre Clinical group Prospective longitudinal	Pre- vs. during CHT	WHOQOL-100	T4 TW: sQoL = 50.25 TM: sQoL = 62.05	None studied	TW reported significant improvement in sexual and general QoL 1 year post-CHT

*BI* Body Image, *CHT* Cross-sex Hormonal Treatment, *Cis* Cisgender, *CRS* Chest Reconstructive Surgery, *GAGS* Gender Confirming Genital Surgery, *LH* Luteinizing Hormone, *sQoL* Sexual QoL, *SR* Social Relationships, *TM* Transgender men, *TW* Transgender women

Regarding predictors of sex-related QoL, CHT [34], low LH in the blood [73], having a partner and experiencing less negative mood symptoms [34] have been found to be factors associated with more positive sex-related QoL.

### Body image-related QoL

Three papers described body image-related QoL, however none of these studies investigated QoL pre-GAT [35, 73, 74]. The cross-sectional study conducted by Castellano et al. [73] found no difference in body image-related QoL between the transgender sample post-medical treatments and a matched cisgender sample. The two longitudinal studies reported an improvement in body image-related QoL after treatment, specifically after CHT [74] and mastectomy for transgender men [35]. This suggests that gender affirming treatments are of benefit to body image-related QoL in transgender samples. The limited research in this area shows that body image related QoL improves post-GAT. See Table 6 for details.

**Table 6:** Studies investigating body image-related quality of life in transgender people (*n* = 3)

Authors (year) Country	Number of Trans participants, mean age at assessment	Treatment status	Study design	Comparative groups, follow-up	Outcome measures	Results	Factors associated	Conclusions
Castellano et al. (2015) [73] Italy	46 TW 14 TM 32.7 yrs. TW 30.2 yrs. TM	+ 2 years post-GAGS 100% CHT 100%	Single centre Clinical group Cross-sectional	60 matched cis control sample (No follow-up)	WHOQOL-100	BodyQoL TW = 64.64 TM = 67.91	+QoL: Lower LH	Trans people reported levels of QoL similar to cis controls
Manieri et al. (2014) [74] Italy	56 TW 27 TM 32.7 yrs. TW 30.2 yrs. TM	T0 = initiation of CHT 100% T1 = 3 months post-CHT 100% T2 = 6 months post-CHT 100% T3 = 9 months post-CHT 100% T4 = 1 year post-CHT 100%	Single centre Clinical group Prospective longitudinal	Pre- vs. during CHT	WHOQOL-100	T4 TW: BI = 21.85 TM: BI = 68.75	None studied	TW reported significant improvement in sexual and general QoL 1 year post-CHT
van de Grift et al. (2016) [34] The Netherlands	26 TM 26.1 yrs	T0: CHT 100% T1: CRS 100% CHT 100%	Single centre Clinical group Prospective longitudinal	Pre- vs. post-CRS (T0 = baseline T1 = 6 months after CRS)	BIQLI	Pre-CRS = 0.32 Post-CRS = 0.38	+QoL: Body satisfaction Feelings of “passing” in social situations	Body satisfaction and “passing” in social situations are associated with higher QoL and self-esteem in TM

*BI* Body Image, *BodyQoL* Body image-related quality of life, *CHT* Cross-sex Hormonal Treatment, *Cis* Cisgender, *CRS* Chest Reconstructive Surgery, *GAGS* Gender Confirming Genital Surgery, *LH* Luteinizing Hormone, *sQoL* Sexual QoL, *SR* Social Relationships, *TM* Transgender men, *TW* Transgender women, *VFT* Voice Feminisation Treatment

Only one study looked at factors associated to body image quality of life and found low levels of LH in the blood to be associated to a positive body image-related QoL [73].

### General (non-condition specific) QoL

Out of the 22 studies that assessed generic (non-condition specific) QoL, there were no cross-sectional studies looking specifically at people pre-GAT. Five studies investigated post-GAT [20, 43, 66, 68, 73], twelve were mixed in term of treatment status [17, 21–23, 42, 54, 55, 65, 69–71, 76], three were longitudinal [56, 64, 74], one did not report information on treatment status [57] and one study reported only that participants were pre-facial feminisation treatment [32].

There is pre-CHT data available from the longitudinal studies showing that transgender people have lower QoL than the general population [56, 74]. Of the six cross-sectional studies looking specifically at people pre-GAGS [21–23, 54, 68, 71], two studies found transgender people to report poorer QoL than the general population [21, 68] and one found similar scores to their matched cisgender controls [22]. The remaining studies did not include comparisons with the general population but they suggested that transgender people report poor QoL.

Five studies investigated QoL at post-GAGS; four studies found that transgender people at this stage still report lower QoL than the general population [43, 66, 68] whilst two studies suggested transgender people to display similar QoL to the general population [20, 73].

With regard to mixed samples, one study suggested transgender people report worse QoL than their matched cisgender controls [69], two reported poorer QoL than the cisgender non-matched controls [21, 70] and one study suggested worse QoL than the general population [65]. Studies looking at people according to their gender found that some of the results regarding QoL in transgender men were contradictory; one study indicated that they suffer from worse QoL than the general population [17] whilst another study suggested the opposite [42]. Transgender men were also found to report a better QoL when undergoing CHT than when not on hormones [71]. Instead, transgender women displayed high physical health-related QoL and poor mental-health related QoL [54]. The remaining three mixed sample studies made comparisons between transgender men and transgender women, and the results are inconsistent. Two studies found that transgender women report better QoL than transgender men [23, 76] and one found no difference between transgender men and transgender women [55]. In another mixed study, Motmans and colleagues [65] found that transgender men had worse QoL than the cisgender population. However the results of the above mixed sample studies need to be interpreted carefully.

Regarding the three longitudinal studies [56, 64, 74], one found an improvement in QoL 1-year post-CHT when compared to pre-CHT levels [74]. A different study also found an improvement in transgender women 1-year post-GAGS when compare to pre-CHT values [56]. The third study compared QoL pre-GAGS (on CHT) and 1, 3 and 5-years post-GAGS. This study found that although QoL pre-GAGS was lower than the general population it improves 1-year post-GAGS. However the study also found that it reduces 3 years post-GAGS and even more 5 years after genital surgery. This could be explained as the first year post-GAGS is often known as the “honeymoon period” and people tend to report overly enhanced QoL, which are not representative of a long-term picture of patients’ psychological status and QoL [43]. When investigating longitudinal results according to gender a study found that transgender women displayed greater improvements in QoL 1-year post-CHT compared to transgender men [74].

Overall, the studies investigating general QoL in transgender people found poorer QoL pre-GAT than the general population, which improve after GAT in the short term. See Table 7 for details.

**Table 7:** Studies investigating general quality of life in transgender people (*n* = 22)

Authors (year) Country	Number of Trans participants, mean age at assessment	Treatment status	Study design	Comparative groups, follow-up	Outcome measures	Results	Factors associated	Conclusions
Ainsworth & Spiegel (2010) [68] USA	247 TW 28 FFS (51 yrs) 28 FFS (51 yrs) 25 GAGS (50 yrs) 47 FFS + GAGS (49 yrs) 147 No surgery (46 yrs)	28 FFS (CHT 86%) 25 GAGS (CHT 100%) 47 FFS + GAGS (CHT 98%) 147 no surgery (CHT 27%)	Clinical group Cross-sectional	CG1 = FFS only CG2 = GAGS only CG3 = FFS + GAGS CG4 = No surgery CG5 = General population (No follow-up)	SF-36-v2	CG1 = 50 CG2 = 49.3 CG3 = 49.2 CG4 = 39.5	+QoL: Surgical treatments	TW have lower QoL than Dutch general female population
Auer et al. (2017) [55] Germany	82 TW 72 TM	TW: CHT 79.3% Pre-GAGS 79.5% TM: CHT 80.6% Pre-CRS 56.9% Pre-GAGS 72.2%	Multicentre (4 sites) Clinical group Cross-sectional	(No follow-up)	SF-36	MCS = 77.66	+QoL: +Sleep quality -Depressive symptoms -Chronic pain (TM) -Anxiety (TW) +Social support (TW) +Body image (TW)	QoL levels did not statistically differ between TW and TM. Substantial portion of low QoL in trans is due to poor sleep quality, anxiety in TW and chronic pain in TM
Başar et al. (2016) [76] Turkey	22 TW 72 TM DMS-IV-TR DSM-V 27.73 yrs. TW 26.82 yrs. TM	CHT: 54.5% TW 20.8% TM; GAGS: 36.4% TW 12.5% TM	Single centre Clinical group Cross-sectional	TW vs. TM (No follow- up)	WHOQOL-BREF-TR	TW = 15.3 TM = 12.7	+QoL: Social support -QoL: Discrimination	Perceived personal discrimination and social support predicted QoL
Bouman et al. (2016) [69] UK	64 TW 40 TM 36.52 yrs	Assessment 6.7% CHT 78.8% 17.3% Post-GCGS	Single centre Clinical group Cross-sectional	140 matched cis control sample (No follow-up)	SF-36-v2	MCS = 70.9	mQoL: Self-esteem Interpersonal issues (too dependent)	Trans people have lower mQoL compared to the cis group
Cardoso da Silva et al. (2016) [56] Brazil	47 TW 21.23 yrs.	T1 at entrance to programme 100% T2 at least 1 year post-GAGS 100%	Single centre Clinical group Prospective longitudinal	Pre- vs. post-GAGS (T1 = baseline T2 = at least 1 year post-GAGS)	WHOQOL-100	T1 = 14.77 T2 = 15.52	+QoL: GAGS	GAGS promotes improvement of psychological aspects of QoL and social relationships, but 1-year post-GAGS TW still report problems with physical health and independence
Castellano et al. (2015) [73] Italy	46 TW 14 TM 32.7 yrs. TW 30.2 yrs. TM	+ 2 years post-GAGS 100% CHT 100%	Single centre Clinical group Cross-sectional	60 matched cis control sample (No follow-up)	WHOQOL-100	TW = 67.87 TM = 69.21	+QoL: Lower LH	Trans people reported levels of QoL similar to cis controls
Colton Meier et al. (2011) [71] USA	369 TM 28 yrs	CHT 66% CRS 41%	Online Cross-sectional	CHT vs. No CHT (No follow-up)	SF-36-v2	hQoL: CHT = 65.2 No CHT = 53.7 Trans = 61.3	+QoL: CHT	CHT is associated with improved mental health in TM
Colton Meier et al. (2013) [42] USA	581 TM 27 years	CHT 67% CRS 41% GAGS 4%	Online Cross-sectional	AM vs. AW vs. AB Normative data (No follow-up)	SF-36-v2	AM = 58.85 AW = 64.77 AB = 60.81	+ QoL: - Depression - Anxiety - Stress + Social Support	TM displayed higher QoL levels than the norm
Davey et al. (2014) [70] UK	63 TW 40 TM 56.9 yrs. TW 28.05 yrs. TM	TW: Post-GAGS 17.5% CHT currently 79.4% TM: Post-GAGS 15% CHT currently 0%	Single centre Clinical group Cross-sectional	Matched cis control sample No follow-up	SF-36-v2	MCS = 69.31	+ MCS, VT, SF QoL: Social support	Trans clinical sample reported lower QoL than matched cis sample
de Vries et al. (2014) [20] The Netherlands	22 TW 33 TM TW: T0 = 13.6 yrs. T1 = 16.5 yrs. T2 = 21 yrs. TM: T0 = 13.7 yrs. T1 = 16.8 yrs. T2 = 20.5 yrs	T0 = pre-puberty suppression T1 = post CHT T2 = 1 year post-GAGS	Single centre Clinical group Prospective longitudinal with cross-sectional data regarding QoL	T0 vs. T1 vs. T2 Participants vs. nonparticipants (T0 = pre-puberty suppression T1 = when CHT introduced T2 = 1 year post-GAGS)	WHOQOL-BREF	T2 pQoL = 14.66	+pQoL: Post-surgical well-being	Well-being in trans same or enhanced compared to same-age general population young adults
Gomez-Gil et al. (2014) [21] Spain	119 TW 74 TM ICD-10 31.2 yrs. Trans	CHT 62.2% No CHT 37.8%	Single centre Clinical group Cross-sectional	101 cis people (No follow-up)	WHOQOL-BREF	pQoL = 56.09	+QoL: CHT Family support Working/studying	Trans reported lower perceived QoL compared to the cis sample. Additionally, TM reported higher social QoL than TW
Gorin-Lazard et al. (2012) [22] France	31 TW 30 TM 39.4 yrs. TW 29.9 yrs. TM	No CHT: TW 19.4% TM 36.7% CHT: TW 80.6% TM 63.3%	Multicentre (3 sites) Clinical group Cross-sectional	French age- and sex-matched control Normative data (No follow-up)	SF-36-v2	MCS = 47.92	+ mQoL: CHT - mQoL: Depression	Positive effect of CHT on QoL. Trans QoL did not differ from cis matched controls except for RP
Gorin-Lazard et al. (2013) [23] France	36TW 31 TM 35.1 yrs. Trans	No CHT: TW 38.9% TM 61.1% CHT: TW 59.2% TM 40.8%	Multicentre (3 sites) Clinical group Cross-sectional	TW vs. TM CHT vs. No CHT (No follow-up)	SQUALA	TW = 12.1 TM = 11.34 Total = 11.72	+ pQoL: CHT	CHT predicted positive self-esteem, less severe depression, and greater psychological dimensions of QoL
Hoy-Ellis et al. (2017) [57] USA	84 TW 51 TM 48 Other 46.88 yrs. TW 27.48 yrs. TM 25.64 yrs. Other	None reported	Online and/or paper Non-clinical group Cross-sectional	Military service vs No military service (No follow-up)	WHOQOL-BREF	pQoL = 64.12	-pQoL: Identity stigma +pQoL: Prior military service	Those with prior military service had lower depressive symptomatology and higher pQoL
Lindqvist et al. (2017) [64] Sweden	T0 = 146 TW T1 = 108 TW T2 = 64 TW T3 = 43 TW 36 yrs	T0 = pre-GAGS + CHT 100% T1 = 1 yr. post-GAGS 100% T2 = 3 yrs. post-GAGS 100% T3 = 5 yrs. post-GAGS	Single centre Clinical group Prospective longitudinal	T0 vs T1 vs T2 vs T3 Swedish normative data	SF-36	MCS: T0 = 73.8 T1 = 74.1 T2 = 71 T3 = 67.6	None studied	TW (both pre and post-GAGS) reported lower QoL than general population; GAGS improves QoL 1 year post-GAGS but it tends to gradually diminish over time
Manieri et al. (2014) [74] Italy	56 TW 27 TM 32.7 yrs. TW 30.2 yrs. TM	T0 = initiation of CHT 100% T1 = 3 months post CHT 100% T2 = 6 months post-CHT 100% T3 = 9 months post-CHT 100% T4 = 1 year post-CHT 100%	Single centre Clinical group Prospective longitudinal	Pre- vs. during CHT	WHOQOL-100	T4 TW: QoL = 63.25 TM: QoL = 72.2	None studied	TW reported significant improvement in sexual and general QoL 1 year post-CHT
Mora et al. (2017) [32] Spain	T0 = 30 TW T1 = 18 TW 30 yrs	Pre-FFS 100%	Single centre Clinical group Prospective longitudinal with cross-sectional data regarding SF12v2	None (No follow-up)	SF-12v2	MCS = 48.63	None studied	Trans women suffer poor QoL
Motmans et al. (2011) [65] Belgium	63 TW 58 TM 42.26 yrs. TW 37.03 yrs. TM	TW: CHT 94.6% FFS 18.7% GAGS 64% TM: CHT 96.7% GAGS 67.8%	Clinical group Cross-sectional	Normative data (No follow-up)	SF-36	MCS = 72.04	+QoL: Being Employed Being in a Relationship Young age, Higher Education Higher household income	TM reported reduced mQoL than Dutch male sample. Older, low educated, unemployed, with a low household income and single trans people had significantly lower QoL
Newfield et al. (2006) [17] USA	376 TM 32.6 yrs	CHT 64% CRS 37% GAGS 11%	Opportunity sampling Cross-sectional	Normative data (No follow-up)	SF-36-v2	MCS = 39.51	+ QoL: Testosterone Usage CRS	TM reported significantly lower mental health-related QoL than US general population
Parola et al. (2010) [66] France	38 Trans 32–65 yrs. range	+2 years CHT and GAGS 100%	Single centre Clinical group Cross-sectional	TW vs. TM; Extraversion vs. Introversion; Neuroticism vs. Emotional stability (No follow-up)	SF-36	TW: Better Social QoL = 11/15 people Better Quality of family relationships = 4/15 people TM: Better Social QoL = 10/15 people Better Quality of family relationships = 6/15 people Extroverted = 54.28 Introverted = 52.02 High neuroticism = 53.16 Low neuroticism = 50.77	+QoL: CHT	TM reported better social and professional QoL, and friendly lifestyles than TW
Wierckx et al. (2011) [43] Belgium	49 TM 37 yrs	Post-GAGS 100% CHT 100%	Single centre Clinical group Cross-sectional	Dutch normative data (No follow-up)	SF-36	MCS = 75.8	QoL: Post-operative sexual functioning	TM have good QoL post-GAGS compared to general Dutch population but still lower than the normative data
Yang et al. (2016) [54] China	209 TW 26.7 yrs	FFS 34.93% CHT 17.70%	Non-clinical group Cross-sectional	None (No follow-up)	SF-36	MCS = 68.28	mQoL: Hope Resilience PhQoL: -Lower age	Chinese TW reported high levels of physical QoL but low levels of mental QoL

*AB* Attracted to Both, *AM* Attracted to Men, *AW* Attracted to Women, *BI* Body Image, *CG* Control Group, *CHT* Cross-sex Hormonal Treatment, *Cis* Cisgender, *CRS* Chest Reconstructive Surgery, *FFS* Face Feminisation Surgery, *GAGS* Gender Confirming Genital Surgery, *hQoL* Health-related QoL, *LH* Luteinizing Hormone, *MCS* Mental Component Score, *mQoL* Mental health-related QoL, *pQoL* Psychological QoL, *p-hQoL* Psychological Health-related QoL, *RP* Role-Physical, *SF* Social Functioning, *sQoL* Sexual QoL, *SR* Social Relationships, *TM* Transgender men, *TW* Transgender women, *VT* Vitality

Medical and surgical treatments (i.e. CHT, CRS, GAGS) [17, 21–23, 56, 66, 68, 71], post-surgical well-being [20] and sexual functioning [43], presence of social and family support [21, 42, 55, 70, 77], decreased depression, anxiety and stress levels [42, 55], lack of chronic pain symptomatology [55], hope and resilience [54], high self-esteem and low levels of interpersonal issues [69], lack of identity stigma [57], having a good body image and good sleep quality [55], low levels of LH in the blood [73] as well as being employed and in a relationship, younger age, higher education, a high household income [65] and having undergone military service [57] were found to be factors predictive of a positive QoL.

## Results of the meta-analysis

### Meta-analysis – Mental health-related QoL of transgender people compared to the general population

Measurements of QoL provide information regarding physical and mental health-related QoL but only a minority of studies looked at physical health-related QoL; therefore the meta-analysis focused on mental health-related QoL compared to those of the general population. Of the 22 studies assessing general QoL in transgender populations, 14 were considered suitable for inclusion in the meta-analysis [17, 20–22, 43, 54–57, 64, 65, 68–70]. These studies include people pre-GAT, post-GAT and mixed groups at different stages of medical transition. Studies were excluded from meta-analysis due to the absence of the mean, SD and/or sample size [66, 74], mental health quality of life not reported separately [73], insufficient detail about scoring [32, 42, 71] and the lack of access to appropriate normative data [23, 76]. Data at pre-treatment were utilised for the two longitudinal studies included in the meta-analysis [56, 64]. Additionally, normative data as comparison was obtained for four studies [54–57].

**Fig. 2:**
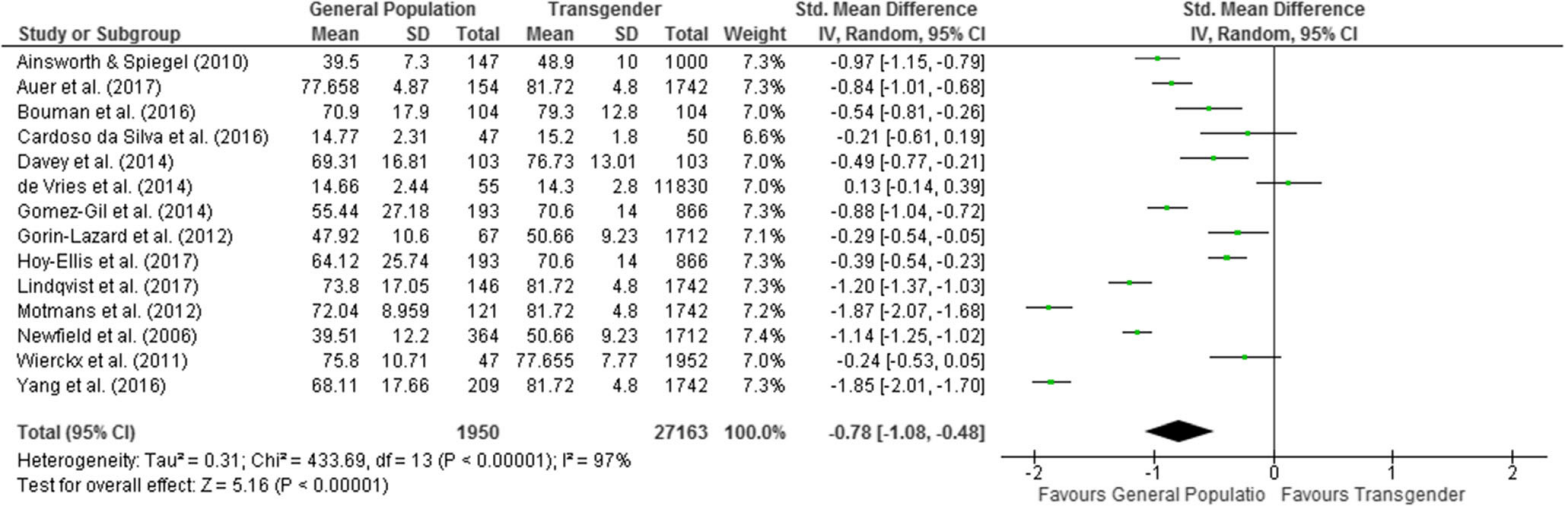
Meta-analysis on mental health-related QoL of transgender people compared to the general population

The results of the meta-analysis (14 studies) showed that transgender people report a statistically significantly lower mental health-related QoL than the general population (standard mean difference − 0.78, 95% CI = −1.08 to −0.48, Z = 5.16, *p* < 0.00001). Heterogeneity was high (*I*^*2*^ = 97%, *p* < 0.00001) (see Fig. 2).

### Meta-analysis – Subgroup analysis – Mental health-related QoL post-hormonal treatment of transgender people compared to the general population

A second meta-analysis was conducted with only the 7 studies that included exclusively post-treatment QoL scores [20, 43, 56, 64, 65, 68, 70]. The longitudinal study of Lindqvist et al. [64] investigated QoL post-GAGS but as the first time measurement was pre-GAGS and thus post-CHT, this measure was included in this analysis. Whilst for the longitudinal study of de Vries et al. [20], values at the latest time-point were used, as they measured QoL post-CHT in a sample of individuals older than 17 years of age.

**Fig. 3:**
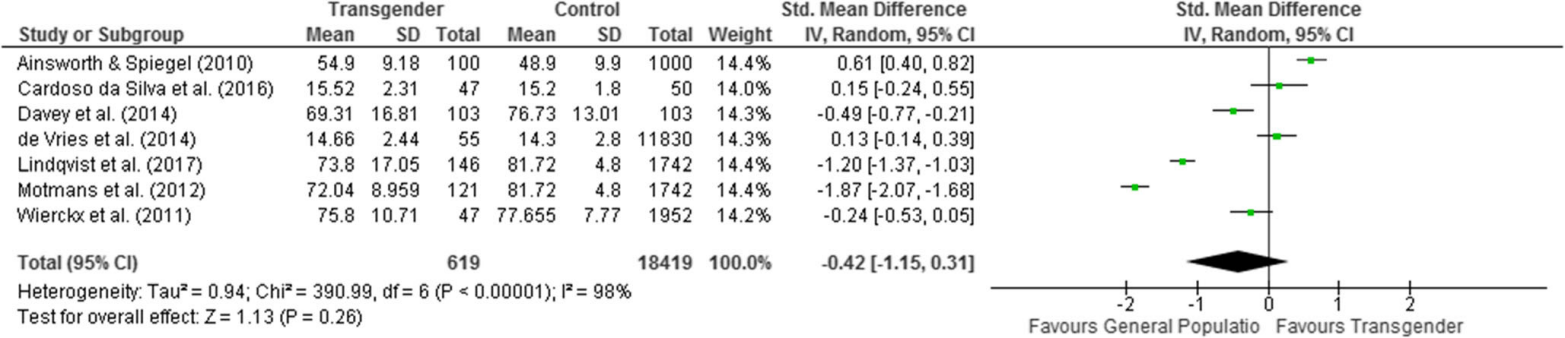
Meta-analysis on mental health-related QoL transgender people post-hormonal treatment compared to the general population

The meta-analysis of 7 studies found that there was no statistically significant difference in mental –health related QoL of transgender people following CHT compared to the general population (standard mean difference = −0.42 CI 95% = −1.15 to 0.31; Z = 1.13; *p* = 0.26). Heterogeneity was high (*I*^*2*^ = 98%; *p* < 0.00001) (see Fig. 3).

## Discussion

The aim of this study was to systematically and critically review the literature pertaining to quality of life in transgender people, to meta-analytically investigate mental-health related QoL compared to cisgender populations and to investigate the impact of GAT on the QoL of this population. A total of 29 studies met the inclusion criteria and were used for the systematic review and, of these, 14 studies were suitable for including in the meta-analysis. Most papers in this area investigated general QoL and only a few focused on either vQoL, sex-related QoL or body image-related QoL. The majority of these articles displayed either high or moderate risk of bias. Many studies used transgender samples that are not homogeneous in terms of gender affirming medical treatment status, which makes it difficult to draw firm conclusions about the impact of GAT.

Findings from the meta-analysis of mental health-related QoL suggest that the QoL of transgender people is significantly poorer than that of the general population, with a medium to large effect size (standard mean difference = 0.78). The subgroup meta-analysis including only the samples of transgender people who were classifiable as post-hormonal treatment found that transgender people post-CHT still had lower mental health-related QoL than the general population. This difference was not significant and the effect size was reduced (standard mean difference = 0.42). The possibility that treatment is associated with improvements in mental wellbeing is supported by the findings from the small number of longitudinal studies in this review. These found that both CHT and GAGS improve QoL [56, 64, 74]. However these results need to be treated with caution, as the only study to employ a longer term follow-up [64] reported that after an initial improvement in QoL at 1-year post-GAGS, scores tend to steadily decrease in the following years until reaching 5-years post-GAGS, when QoL is lower than at pre-treatment [64]. During the first year post-GAGS people tend to report overly enhanced QoL, which may not be representative of a long-term picture of patients’ psychological status and QoL [43]. The improvement in QoL experienced by transgender people at short-term could be attributed to relief at being able to live as the experienced gender. Additionally, as QoL in the general population has been shown to decrease with age [53], a decline in these scores is somewhat expected as time passes.

In contrast, the small number of studies that explore general physical health-related QoL suggest that at post-GAT, transgender people’s reported QoL scores either similar to [22] or better than that found in the general population [17, 43]. However, only a minority of studies report findings related to physical health-related QoL and it is difficult to draw accurate conclusions.

### Condition specific QoL

When looking at condition-specific QoL, studies investigating vQoL reported that CHT has been shown to have a positive impact on transgender men. This is not surprising, as testosterone is known to affect voice by thickening vocal chords and by decreasing the pitch [87]. On the other hand, studies in transgender women, including post-voice feminising surgery, found that they still feel handicapped regarding their voice in their everyday life, irrespective of the transitional status. In fact, studies have suggested that the more feminine a transgender person perceives her own voice, the higher the experienced vQoL [30, 31]. However, these studies are limited by focusing on transgender women who transitioned post-puberty. This means that by the time they initiated physical transition, testosterone has already negatively altered their voice. Thus findings from vQoL cannot be generalised to the overall transgender population. Future studies should explore differences in vQoL between those who transitioned pre-puberty and therefore before the breaking of the voice, and those who transitioned post-puberty, when the voice has already been affected.

Regarding sex-related QoL, longitudinal studies suggest that undergoing GAT improves sex-related QoL [74] but the QoL of transgender men post-GAGS is still worse than that of cisgender men. However, the articles investigating sex-related QoL in transgender men did not distinguish whether patients underwent phalloplasty or metoidioplasty. Surgical treatments help the transgender person reach the desired physical changes and lead towards a more congruous body with their gender identity. This may lead to people feeling more comfortable with their own bodies and consequently when being intimate with others.

Longitudinal studies also supported an amelioration in body image-related QoL [35, 74]. In fact, Castellano and colleagues [73] reported no difference between the transgender population and their matched controls. This could be due to the fact that people undergoing GAGS are generally already on CHT and hormonal treatment is known to have a positive effect on body image [10, 36, 88] by aiding in the development of desired secondary sexual characteristics of the experienced gender, whilst helping to alter some of the attributes relative to the sex assigned at birth. Consequently, this leads to an improvement in body image-related QoL. Nonetheless, van de Grift et al. [35] proposed body image-related QoL to be lower for transgender people post-CRS, than for the general population. This might be caused by the fact that following CRS, some people’s genital dysphoria may increase. However, caution is still needed when generalising the findings due to the studies’ moderate risk of bias as well as their methodological limitations.

### General QoL

Studies regarding general QoL have found that transgender people’s QoL is poorer than that of cisgender people, but that it improves post-GAT. The poorer QoL found in the transgender population pre-CHT [56, 74] could be explained by the high degree of mental health problems reported in this population [16, 89] as well as by the difficulties that many have in socialising and living a fulfilling life [10–12, 37]. However, the studies that focus only on people pre-CHT were rare and only included those seeking medical transition, which does not allow for a generalisation of these findings to the general transgender population.

When looking at general QoL post-GAT, only a small number of studies provided control data and none of them had a low risk of bias. Findings of the subgroup meta-analysis at post-treatment showed that there is no difference in general QoL between transgender people and the general population. The improvement in QoL post-GAT could be due to the effect of treatment in the reduction of dysphoria and mental health problems, such as self-harm and depression [13, 90].

Overall, findings support the idea that QoL improves following hormonal treatment [74] as well as post-genital surgeries [43, 56]. As people undergoing surgery are generally already on hormones, the exact role of genital surgery in QoL cannot be extrapolated from these studies. Often, even after an improvement in QoL post-surgical treatment, transgender people reported lower QoL compared to cisgender individuals [7, 56, 64, 70]. This could be due to the fact that, even if being happier with their own bodies, society is still not ready to accept transgender people; thus work, education or relationships can be affected by being transgender [6, 91, 92]. This is confirmed by the findings of studies investigating factors predictive of QoL in this population, as described in the section below.

Caution is needed while interpreting the results in comparison to cisgender individuals as not all studies have matched controls and sample sizes are generally small. Additionally, as the majority of the studies investigated QoL in transgender clinical populations, generalisation of findings for the general transgender population is hindered.

Studies on differences between transgender men and transgender women advance contrasting results. Two studies seemed to suggest that transgender men display lower QoL compared to transgender women [23, 76], two studies suggested the opposite [21, 66], whilst one study proposed no statistically significant differences between groups [55]. Literature also suggested that at 2-years post-treatment transgender men display higher QoL than transgender women [66], whilst still lower than cisgender people [73]. These findings might be due to baseline differences in QoL scores [65] as well as because of the utilisation of mixed samples in terms of treatment status. Results need to be interpreted with caution as none of the articles displayed a low risk of bias. A possible explanation for transgender men to report higher QoL than transgender women might be due to the wider social acceptance towards masculinity than femininity. This presents itself with transgender men reporting less marked psychopathology, getting involved more easily in society and being employed in more stable jobs, whilst feeling less limitations in daily life related to their physical and emotional state [21, 66]. Additionally, studies that reported transgender women to display higher QoL than transgender men suggested that these findings are unexpected and surprising [76] considering the low social status of and amount of discrimination faced by transgender women in some countries (e.g. Turkey).

### Factors associated with QoL

QoL can be influenced by a wide array of factors, which can predict both its increment, as well as its decline. Literature looking at variables associated with a positive QoL for transgender people suggested that undergoing medical and surgical treatments (i.e. CHT, CRS, GAGS) are the main predictive factors, irrespective of the QoL domain studied [17, 21–23, 34, 66, 68, 71]. These findings were confirmed by longitudinal studies, which indicated an improvement in QoL from pre- to post-treatment [35, 56, 74].

Additionally, social and family support, being employed, being in a relationship, being younger, having a partner, being highly educated, having a high household income, and the presence of past military service were associated with improved scores on general and sex-related QoL [21, 34, 54, 57, 65].

Instead, anxiety, poor sleep quality, experiencing pain, reduced self-esteem and high interpersonal issues are factors that have been linked to poor QoL in both transgender populations [22, 42, 55, 69, 70, 76] as well as in the general population [93].

## Conclusion

As all systematic literature reviews, this study is also limited by the amount and quality of the published literature available. Future studies should employ more robust methodologies, which explore QoL in a more homogeneous population and using matched control groups.

Despite the limitations of the published literature, this review concludes that overall transgender people display poorer QoL than the general population, particularly pre-GAT, and that QoL improves once people are on CHT.

When specifically looking at the different dimensions of QoL (vQoL, sex-related QoL, and body image-related QoL), findings of the systematic review suggest that transgender people display poorer QoL than the general population, independent of the QoL domain investigated. As per general QoL, all dimensions of QoL have been shown to improve post-GAT. However, as the effect of GAT is linked to gender, a more positive vQOL was found for transgender men than transgender women at post-GAT, whilst opposite findings were obtained for sex-related QoL.

As long-term follow-up studies are limited in numbers and methodology, more studies are required exploring long-term QoL. This information may aid the development of support and interventions aiming at increasing resilience for those at risk of a poor QoL post-GAT.
